# Fast Detection of a *BRCA2* Large Genomic Duplication by Next Generation Sequencing as a Single Procedure: A Case Report

**DOI:** 10.3390/ijms18112487

**Published:** 2017-11-22

**Authors:** Marcella Nunziato, Flavio Starnone, Barbara Lombardo, Matilde Pensabene, Caterina Condello, Francesco Verdesca, Chiara Carlomagno, Sabino De Placido, Lucio Pastore, Francesco Salvatore, Valeria D’Argenio

**Affiliations:** 1 CEINGE-Biotecnologie Avanzate, via Gaetano Salvatore 486, 80145 Naples, Italy; nunziato@ceinge.unina.it (M.N.); starnone@ceinge.unina.it (F.S.); barbara.lombardo@unina.it (B.L.); verdesca@ceinge.unina.it (F.V.); lucio.pastore@unina.it (L.P.); 2 Department of Movement Sciences and Wellness (DiSMEB), University of Naples Parthenope, via Medina 40, 80133 Naples, Italy; 3 Department of Molecular Medicine and Medical Biotechnologies, University of Naples “Federico II”, via Sergio Pansini 5, 80131 Naples, Italy; 4 Oncology Division, Department of Clinical Medicine and Surgery, University of Naples “Federico II”, 80131 Naples, Italy; matrod@libero.it (M.P.); catecond@yahoo.it (C.C.); 5 Department of Clinical Medicine and Surgery, University of Naples “Federico II”, 80131 Naples, Italy; chiara.carlomagno@unina.it (C.C.); sabino.deplacido@unina.it (S.D.P.); 6 IRCCS-Fondazione SDN, via Emanuele Gianturco 113, 80143 Naples, Italy

**Keywords:** hereditary breast cancer, *BRCA2*, large genomic rearrangements, next generation sequencing, aCGH

## Abstract

The aim of this study was to verify the reliability of a next generation sequencing (NGS)-based method as a strategy to detect all possible *BRCA* mutations, including large genomic rearrangements. Genomic DNA was obtained from a peripheral blood sample provided by a patient from Southern Italy with early onset breast cancer and a family history of diverse cancers. *BRCA* molecular analysis was performed by NGS, and sequence data were analyzed using two software packages. Comparative genomic hybridization (CGH) array was used as confirmatory method. A novel large duplication, involving exons 4–26, of *BRCA2* was directly detected in the patient by NGS workflow including quantitative analysis of copy number variants. The duplication observed was also found by CGH array, thus confirming its extent. Large genomic rearrangements can affect the *BRCA1/2* genes, and thus contribute to germline predisposition to familial breast and ovarian cancers. The frequency of these mutations could be underestimated because of technical limitations of several routinely used molecular analysis, while their evaluation should be included also in these molecular testing. The NGS-based strategy described herein is an effective procedure to screen for all kinds of *BRCA* mutations.

## 1. Introduction

Germline mutations in the *BRCA* genes confer an increased risk for breast and ovarian cancers [1]. In fact, about 15% of all breast cancer cases fall within the hereditary breast and ovarian cancer (HBOC) syndrome, and *BRCA1* and *BRCA2* are the two major HBOC susceptibility genes identified so far [2]. *BRCA* mutations carriers have a higher lifetime risk of different types of cancers, which often occur at an earlier age with respect to the general population [1,2]. Consequently, carriers’ early identification is important to offer patients and their families the most appropriate surveillance screening and prophylactic, therapeutic or surgical options [3,4,5]. Given the large size of both *BRCA1* and *BRCA2* genes and the absence of mutational hot spots, highly sensitive methods are required for the molecular analysis of the *BRCA* genes [6]. Most of the known *BRCA1/2* mutations are single nucleotide substitutions or small insertions/deletions (https://research.nhgri.nih.gov/projects/bic/), easily detectable by DNA sequencing methods. However, generally these methods are not able to identify large genomic rearrangements (LGRs) [7]. 

LGRs, including deletions, duplications or insertions larger than 500 kb, have been identified in the *BRCA* genes, with a frequency between 0 and 28% depending on the population analyzed [7,8,9,10,11]. These mutations appear to be more frequent in the *BRCA1* gene, probably due to the higher frequency of *Alu* sequences in this gene and/or due to homologous recombination events between *BRCA1* and its pseudogene [7,8,9,10,11]. To date, various methods have been used to identify LGR in the *BRCA* genes, namely Southern blot, quantitative PCR, and multiplex ligation probes amplification [7,8,9,10,11]. Thus far, 81 and 24 different LGRs have been reported in *BRCA1* and *BRCA2*, respectively [9,12]. Consequently, LGRs account for a not negligible proportion of *BRCA* mutations, and their investigation should be included in *BRCA* genes molecular analysis workflow.

Thanks to advances made in next-generation sequencing (NGS) methods, these procedures have shown their potentiality for molecular analysis at DNA level and are now widely used in the clinical setting, such as for *BRCA* genes testing [6,13,14,15,16]. Since these technologies generate millions of sequences of the same target genomic region, it is likely that, coupled with the appropriate bioinformatics tools, the analysis of quantitative sequencing reads can be used to estimate LGRs [17,18]. 

Here, we report a large duplication of the *BRCA2* gene identified in an Italian HBOC family by NGS and confirmed by array-based comparative genomic hybridization (aCGH). Our findings indicate that NGS-based methods can be an effective strategy with which to analyze *BRCA1/2* mutations, which also include LGRs. The large-scale use of this approach will lead possibly to an increase in the number of LGRs in *BRCA1/2* genes, and to better assess their contribution to the risk of HBOC predisposition in predictive medicine, which is also appropriate for medical decision.

## 2. Results

**Figure 1:**
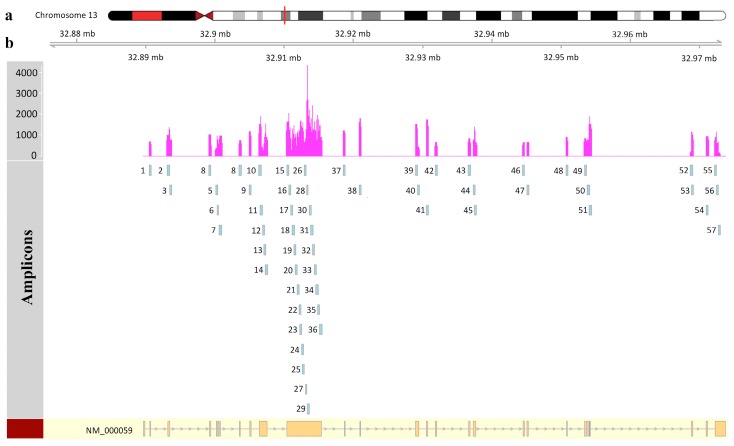
Next generation sequencing coverage of the *BRCA2* gene. (**a**) The relative position of the *BRCA2* gene on chromosome 13. (**b**) The patient’s coverage for each sequenced amplicon is reported with respect to *BRCA2* exons at the bottom.

In the NGS-based analysis of the *BRCA* genes, we sequenced 69,979 read pairs, of which 69,883 (corresponding to 99.86% of all reads) were successfully mapped. We did not find any low coverage regions, defined as regions that fall within amplicons but have a coverage less than 50X (Figure 1).

**Figure 2:**
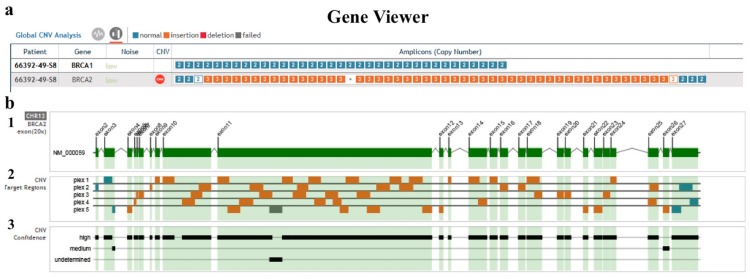

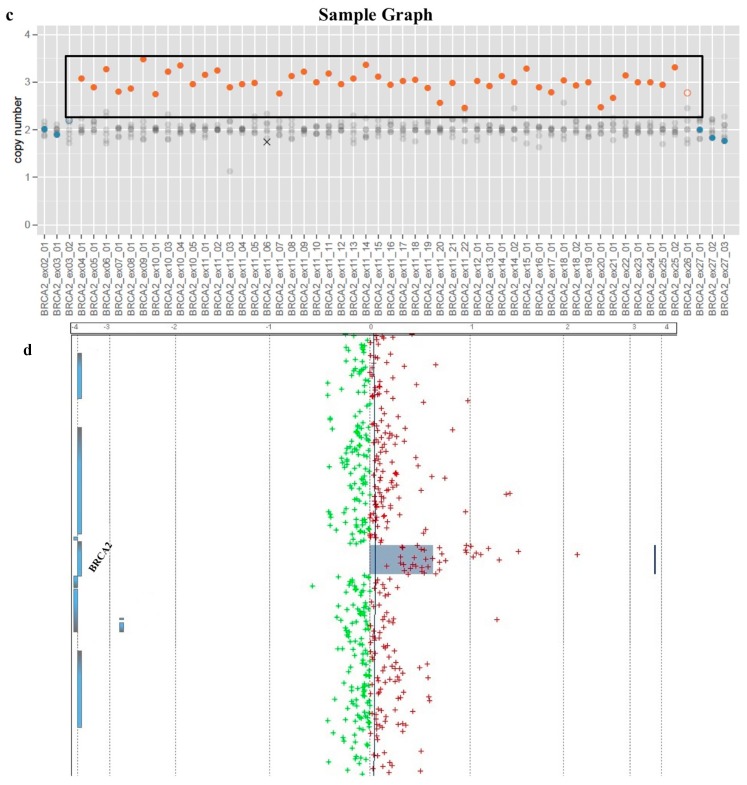
Large *BRCA2* gene duplication. (**a**) Gene view of the global copy number variant (CNV) analysis of the *BRCA1* and *BRCA2* genes. The noise is low for both genes. *BRCA1* contains no CNVs in any of the target amplicons, while *BRCA2* presents a large duplication, from exon 4 to exon 26. (**b**) The CNV analysis of the *BRCA2* gene. b1 *BRCA2* with all the exons; b2 CNV target regions for all the 5 plexes of the MASTR BRCA DX (Multiplicom); b3 CNV confidence plot showing a high confidence for almost all target amplicons. (**c**) Sample graph for each amplicon in *BRCA2* showing the large duplication. There is an undetermined region for the amplicon 11_06 probably due to a region difficult to analyze. (**d**) Array-based comparative genomic hybridization (a-CGH) profile of chromosome 13. This analysis shows heterozygous amplification in 13q13.1 of about 72 kb involving the *BRCA2* gene. Green and red dots represent the log2 fluorescence ratios of individual oligonucleotide probes on the microarrays; red dots represent probes with positive log2 fluorescence ratios, whereas green dots represent probes with negative log2 fluorescence ratios.

Furthermore, the sequence coverage found in all our amplicons belonging to our patient was at least 100X for all the regions. Both the software we used confirmed the absence of pathogenic point mutations in our patient. Instead, the CNV analysis with the second described software highlighted a low-noise status and a very large duplication (duplication ratio = 3) in high-confidence status in the *BRCA2* gene (13: 32,899,089-32,971,294) involving about 72,205 bp (Figure 2a–c).

In the *BRCA2* region predicted to be duplicated, we identified 8 SNPs: 3 were homozygous while the other 5 were present in heterozygous status (Supplementary Materials Table S1). Usually, in a wild-type diploid asset, the reads distribution for a heterozygous variant ranges from 45% to 55% of all the obtained reads covering that variant. This corresponds to the fact that one allele carries the variant and the other does not. In our patient, no one of the five variants detected as heterozygous has this kind of frequency: 3 have a frequency higher than expected, ranging from 64–67%, and 2 have a frequency lower than expected (36–37%). This finding supports the presence of the duplicated allele: assuming that we are simultaneously analyzing 3 alleles, each of them accounts for about 33% of all the reads. Thus, variants occurring on the wild type allele will have a frequency of about 33%, while variants occurring in the duplicated allele will have a frequency of about 66%: these are exactly the values we found in our patient.

As shown in Figure 2d, aCGH analysis of our patient highlighted a heterozygous amplification on chromosome 13 at the q13.1 region of the *BRCA2* gene, namely, chr13.hg19:g. (32,897,658-32,897,717)–(32,969,954-32,970,004) amp (range between 72,296 and 72,287 bp). The exact breakpoints of the duplication insertion should be searched in detail by other techniques.

## 3. Discussion

Here, we report the identification of a large novel *BRCA2* duplication in an HBOC patient obtained using an NGS-based method. The tested patient is a 43-year-old woman from Southern Italy who had an early onset breast cancer (under 40 years of age). Since she has a family history suggestive of hereditary cancer, after genetic counseling, she underwent *BRCA* molecular testing to verify her mutational status. We previously assessed the efficacy of an NGS-based approach for the fast detection of *BRCA* point mutations and small insertions/deletions [6]. Since, in principle, NGS can be used also for quantitative analysis and, thus, overcome a typical limitation of DNA sequencing analysis by detecting also LGRs, we assessed this hypothesis in our case report. In our patient, no pathogenic point mutations were identified in *BRCA1* or in *BRCA2*. Instead, bioinformatics analysis of LGRs revealed a large duplication in *BRCA2* involving exons 4–26, whose presence was confirmed by a-CGH analysis. Hitherto, *BRCA* LGRs have been considered rare events and, only a few *BRCA2* LGRs have been characterized [7,8,9,10,11,12,17]. 

Our data reinforce the concept that the frequency of *BRCA* LGRs is probably underestimated since they elude currently used diagnostic strategies, which include only point mutations or small indels [7,8,9,10,11,12,17]. Furthermore, other methods for CNV detection require additional diagnostic workflows, thereby increasing the time to results and also the costs of the molecular tests. Here, we have assessed the reliability of a NGS-based *BRCA* genes analysis to simultaneously detect most of the possible pathogenic mutations affecting these genes, including LGRs. Indeed, the possibility to have a unique method for the fast and accurate detection of all the possible genetic variants affecting a gene of interest, including LGRs, is a goal of molecular diagnostics, also in the field of *BRCA* genes evaluation. Consequently, this is actually a very hot topic since previous studies lack data regarding LGRs detection [7,8,9,10,11,12,17]; certainly, NGS give the opportunity to fill-in this gap. Even if we report just one case, we believe that our results reinforce the need to include LGRs screening in the *BRCA* routine assessment on one side. In addition, we describe a very rapid and accurate procedure and, finally and not less important, we also report a novel, not previously detected large *BRCA2* duplication. The results obtained in our case report demonstrate that such a NGS-based analysis could serve as a cancer risk prediction tool able to detect point mutations and small insertions/deletions, and also to highlight those patients carrying known and unknown LGRs. This strategy not only optimizes laboratory settings since only one molecular workflow is required, but, by enlarging the spectrum of mutations detected, also increases the diagnostic sensitivity of the molecular test. Finally, the application of this strategy in large-scale population studies will shed light on the frequency of *BRCA* LGRs in HBOC patients.

### Note after Discussion

While this paper was under our internal revision, Concolino et al. [19] reported a large duplication in *BRCA2*, not very different to the novel one we describe in this manuscript. We plan to study our case report in greater depth to evaluate the possibility that this duplication may occur in an easier mutational event area. It is noteworthy that our results were obtained using a different methodological NGS approach with a pipeline analysis of a single workflow that results in a fast turn-around time and with a bioinformatics tool that simultaneously analyzes sequences for CNVs evaluation.

## 4. Materials and Methods

### 4.1. Enrollment of Patients and Collection of Samples

**Figure 3:**
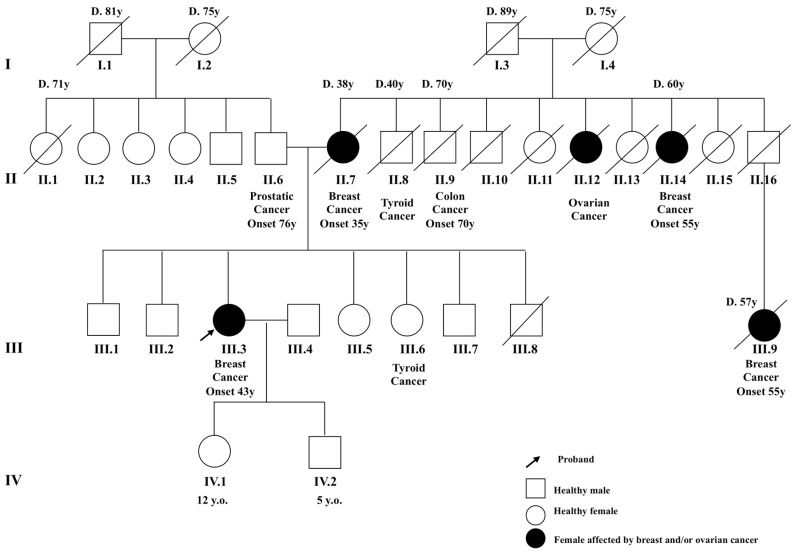
Pedigree of the patient. Our patient (III.3) is indicated by the arrow. Breast, ovarian, colon and prostate cancers are reported in the family. D.: age of death; y.o.: years old.

The patient, now 43 years old, was enrolled from among those attending the Oncology Division, Department of Clinical Medicine and Surgery, of the University of Naples “Federico II”, Naples, Italy. She was diagnosed with early onset breast cancer and had a positive family history for breast, colon, prostate and ovarian cancers (Figure 3).

According to the National Comprehensive Cancer Network guidelines [20], pre-test genetic counseling was carried out to evaluate the familial risk for HBOC and suggested molecular analysis be performed based on patient’s personal and familial history. All clinical data were collected and a three-generation family pedigree was constructed to report the number and the type of cancers present in the family (Figure 1). The patient gave her written informed consent to the molecular test. To date, the other unaffected members of the family, including the sisters and the brothers of the patient described herein, were not available for the molecular analysis to assess the presence of the *BRCA2* familial mutation.

Genomic DNA was isolated from a peripheral blood sample using the Maxwell^®^ 16 LEV Blood DNA Kit (Promega Corporation, Madison, WI, USA) according to the manufacturer’s instructions, and eluted in molecular biology grade pure water. DNA quantity was evaluated using the NanoDrop 2000c Spectrophotometer (Thermo Fisher Scientific, Waltham, MA, USA), while DNA quality was assessed by 0.8% agarose gel electrophoresis.

### 4.2. Next Generation Sequencing

The NGS analysis of the *BRCA* genes is described elsewhere [6]. Multiple amplicon DNA library, covering all the *BRCA1* and *BRCA2* coding and flanking intronic regions, was obtained using the BRCA MASTR Dx Assay kit (Multiplicom, Niel, Belgium), according to the manufacturer’s instructions. Sequencing reaction was carried out using the reagent kit V2 nano, 2 × 250PE, on the Illumina MiSeqSystem (Illumina Inc. San Diego, CA, USA), according to the manufacturer’s instructions.

### 4.3. Bioinformatic Analysis

The sequencing data were analyzed with two bioinformatics software: the SeqPilot software version 4.2 (JSI Medical Systems GmbH, Kippenheim, Germany) was used to detect point mutations, and the Sophia DDM^®^ software version 4.2 (Sophia Genetics SA, Saint Sulpice, Switzerland) was used to confirm point mutations and to identify LGRs. 

The SeqNext module of the Sequence Pilot detects the variants present in each sample by mapping the sequence reads against *BRCA1* and *BRCA2* reference sequences (the ENSG00000012048 gene reference and ENST00000357654.7 transcript from the Ensemble Database for *BRCA1*, and the ENSG00000139618 gene reference and the ENST00000380152.7 transcript from the Ensemble Database for *BRCA2*). The SeqNext module sorts the reads for each sample according to their index, and a variant is called only if it is present in more than 15% of combined reads (both in forward and reverse strands). If a variant occurs within a homopolymer (a sequence region with 6 or more repeats of the same nucleotide), it is called only if its frequency exceeds 20% of reads. The identified variants were characterized and classified according to their in silico gene function predictions and biological significance using the Ensembl (http://www.ensembl.org/) and ClinVar (https://www.ncbi.nlm.nih.gov/clinvar/) databases. 

In addition to point mutation analysis, the Sophia DDM^®^ software is also used to identify copy number variants (CNVs) for LGR detection. In fact, it analyzes the coverage levels of the target regions across all the samples that have been sequenced together. For each sample, the algorithm automatically selects a set of reference samples that have similar coverage patterns in the same run. Based on the reference samples, coverage is normalized by sample and by the target region, and CNVs are called using a hidden-Markov-model algorithm, and thus the most probable copy-number for each target region is determined. In presence of a wild type diploid asset a value of “2” is assigned to each amplicon referring to the two alleles of autosomal genes. In the case of a deletion, corresponding to the loss of one allele, a value of “1” will be assigned. Similarly, in the presence of a duplication a value of “3” will be assigned to the duplicated amplicon referring to the additional copy. A confidence level is determined at both sample and the target-region level. Samples are classified into “rejected”, “medium-noise” and “low-noise” based on the residual coverage noise after normalization and CNV calling. No CNV results are reported for rejected samples, but plots of the coverage profile of those samples are available in the analysis output. After the analysis of CNVs, each amplicon in non-rejected samples are also classified into three categories: “high-confidence”, “medium-confidence” and “undetermined”. 

At the end of each data analysis, both software generate a report for each patient The SeqPilot report shows point mutations and small indels, and contains information about the sequence-coverage per exon. The Sophia DDM report shows the list of retained variants classified in 4 groups according to their clinical significance (A, Most likely pathogenic; B, Potentially pathogenic; C, Variants of unknown significance; and D, Most likely benign), and a CNV Table of the LGRs detected and indications of genomic coordinates (chromosome, start, stop) and type (deletion or duplication).

### 4.4. Comparative Genomic Hybridization Array

A high resolution a-CGH analysis was performed on the patient’s genomic DNA. Briefly, genomic DNA was digested, labeled and hybridized on the surface of the SurePrint G3 Human CGH Microarray Kit, 1 × 1 M with 2.1 kb overall median probe spacing (1.8 kb in Refseq genes), according to the manufacturer’s protocols already reported above (Agilent Technologies, Santa Clara, CA, USA). Then, the microarray was scanned on an Agilent G2600D scanner, and image files were quantified using Agilent’s Feature Extraction software (V11.5.1.1); data were visualized with Agilent’s Genomic Work Bench Standard Edition (V7.0.4.0).

## Supplementary Materials

Supplementary Materials can be found at www.mdpi.com/1422-0067/18/11/2487/s1.

## Abbreviations

HBOC	hereditary breast and ovarian cancer
LGR	large genomic rearrangement
NGS	next generation sequencing
aCGH	array-based comparative genomic hybridization
CNV	copy number variant
