# Supplementary material for: Fast Detection of a BRCA2 Large Genomic Duplication by Next Generation Sequencing as a Single Procedure: A Case Report
Source: Int J Mol Sci. 2017 Nov 22;18(11):2487. doi: 10.3390/ijms18112487 (PMC5713453; doi:10.3390/ijms18112487)
Supplement: Supplementary file 1 [file ijms-18-02487-s001.pdf]

**Supplementary Table S1.** Sequencing report of all the *BRCA2* SNPs found in our patient.

| Exon number | Reference SNP ID | cDNA | Protein* | Genotype status | Variant Coverage (reads number) |
|---|---|---|---|---|---|
| 8 | rs2126042 | c.681+56C>T | - | Het | 64% (930) [64% (470) - 64% (460)] |
| 10 | rs144848 | c.1114A>C | p.N372H | Het | 37% (277) [37% (136) - 37% (141)] |
| 10 | rs530328582 | c.1909+22delT | - | Het | 36% (317) [0% (0) - 36% (317)] |
| 11 | rs206075 | c.4563A>G | p.L1521= | Hom | 100% (1920) [100% (970) - 100% (950)] |
| 11 | rs206076 | c.6513G>C | p.V2171= | Hom | 100% (2231) [100% (1088) - 100% (1143)] |
| 14 | rs169547 | c.7397T>C | p.V2466A | Hom | 99% (796) [100% (393) - 99% (403)] |
| 17 | rs9534262 | c.7806-14T>C | - | Het | 67% (562) [67% (562) - 0% (0)] |
| 22 | rs4942486 | c.8755-66T>C | - | Het | 67% (551) [67% (551) - 0% (0)] |

SNP: single nucleotide polymorphisms; Het: heterozygous; Hom: homozygous.

*According to HGVS nomenclature guidelines
